# CNS involvement in OFD1 syndrome: a clinical, molecular, and neuroimaging study

**DOI:** 10.1186/1750-1172-9-74

**Published:** 2014-05-10

**Authors:** Ennio Del Giudice, Marina Macca, Floriana Imperati, Alessandra D’Amico, Philippe Parent, Laurent Pasquier, Valerie Layet, Stanislas Lyonnet, Veronique Stamboul-Darmency, Christel Thauvin-Robinet, Brunella Franco

**Affiliations:** 1 Department of Translational Medical Sciences, Federico II University of Naples, Naples, Italy; 2 Telethon Institute of Genetics and medicine (TIGEM), Via Pietro Castellino 111, 80131 Naples, Italy; 3 Department of Radiological Sciences, Neuroradiology Unit, Federico II University of Naples, Naples, Italy; 4 Service de Pédiatrie et de Génétique, CHU, Brest, France; 5 Service de Génétique Médicale, Centre de Référence Maladies rares Labélisé « Anomalies de Développement et Syndromes Malformatifs » de l’Ouest, CHU Rennes, Rennes, France; 6 Consultation de Génétique, Groupe Hospitalier du Havre, CH Le Havre, Le Havre, France; 7 Département de Génétique, Hôpital Necker-Enfants Malades, APHP, Paris, France; 8 Service de Pédiatrie 1, Hôpital d’Enfants, CHU Dijon, Dijon, France; 9 Centre de Génétique et Centre de Référence Maladies rares Labélisé « Anomalies de Développement et Syndromes Malformatifs » de l’Est, FHU_TRANSLAD, Hôpital d’Enfants, Dijon, France; 10 EA 4271 GAD « Génétique des Anomalies du développement », IFR 100 - Santé STIC, Université de Bourgogne, Dijon, France

**Keywords:** OFD1, Ciliopathies, Neuroimaging, Neurodevelopmental phenotype

## Abstract

**Background:**

Oral-facial-digital type 1 syndrome (OFD1; OMIM 311200) belongs to the expanding group of disorders ascribed to ciliary dysfunction. With the aim of contributing to the understanding of the role of primary cilia in the central nervous system (CNS), we performed a thorough characterization of CNS involvement observed in this disorder.

**Methods:**

A cohort of 117 molecularly diagnosed OFD type I patients was screened for the presence of neurological symptoms and/or cognitive/behavioral abnormalities on the basis of the available information supplied by the collaborating clinicians. Seventy-one cases showing CNS involvement were further investigated through neuroimaging studies and neuropsychological testing.

**Results:**

Seventeen patients were molecularly diagnosed in the course of this study and five of these represent new mutations never reported before. Among patients displaying neurological symptoms and/or cognitive/behavioral abnormalities, we identified brain structural anomalies in 88.7%, cognitive impairment in 68%, and associated neurological disorders and signs in 53% of cases. The most frequently observed brain structural anomalies included agenesis of the corpus callosum and neuronal migration/organisation disorders as well as intracerebral cysts, porencephaly and cerebellar malformations.

**Conclusions:**

Our results support recent published findings indicating that CNS involvement in this condition is found in more than 60% of cases. Our findings correlate well with the kind of brain developmental anomalies described in other ciliopathies. Interestingly, we also described specific neuropsychological aspects such as reduced ability in processing verbal information, slow thought process, difficulties in attention and concentration, and notably, long-term memory deficits which may indicate a specific role of OFD1 and/or primary cilia in higher brain functions.

## Background

Primary cilia are microtubule-based organelles protruding from the surface of most mammalian cells, including neuronal cells, which exert mainly sensory functions [1]. Human disorders associated with ciliary dysfunction are called “ciliopathies” and present phenotypes that vary from renal and hepatic cystic disease to neurologic phenotypes, disorders of laterality, obesity, and skeletal disorders. In particular, central nervous system (CNS) involvement includes intellectual disability and a wide range of brain structural abnormalities [2].

To date various ciliopathies have been identified, including pleiotropic disorders such as the OFD type 1 (OFD1), Bardet-Biedl syndrome (BBS), Joubert syndrome and related disorders (JSRDs), Meckel-Gruber syndrome (MKS) [1].

OFD type 1 syndrome (OFD1; OMIM 311200) is a rare X-linked-dominant male-lethal developmental disorder which was first described by Papillon-Leage and Psaume in 1954 [3]. The disease belongs to the heterogeneous group of disorders known as oral–facial–digital syndromes (OFDs) [4,5]. OFD1 is characterized by malformations of the face, oral cavity, and digits with a high degree of intrafamilial and interfamilial phenotypic variability possibly due to X chromosome inactivation [6]. CNS involvement is reported in 60% of cases and includes brain structural abnormalities, intellectual disability and/or selective cognitive impairment. Renal cystic disease is reported in the majority of cases occurring in patients older than 18 [5,7-9]. OFD type 1 syndrome is caused by mutations in the *Cxorf5* transcript, subsequently named *OFD1*[10,11]. To date, 130 different mutations (9 genomic deletions and 121 point mutations, mostly represented by frameshifts resulting in truncating forms of the protein) have been identified in OFD type 1 cases. Over 70% of patients represent sporadic cases, and no clear genotype/phenotype correlation has been established (reviewed in [5] and [9]). Interestingly OFD1 has also been found mutated in a X-linked recessive intellectual disability syndrome comprising macrocephaly and ciliary dysfunction [12], in X-linked Joubert syndrome [13-16], and more recently in X-linked retinitis pigmentosa [17]. Retinitis pigmentosa is not commonly found in OFD type 1 as often as the recurrent respiratory tract infections described in Budny et al. On the contrary there are significant similarities between the CNS phenotype described in JSRDs and OFD type 1 (see discussion). To date no clear genotype-phenotype correlation has been established concerning the involvement of OFD1 in these different genetic conditions.

The *OFD1* gene encodes a centrosomal/basal body protein localized at the base of primary cilia [18,19]. Characterization of *in vitro* (*Ofd1*-silenced cells and ES cells depleted for the *Ofd1* transcript) and *in vivo* (null mutants) models demonstrated that, similar to what is described for other ciliary proteins, inactivation of the *Ofd1* transcript is associated with defective Sonic hedgehog (Shh) and canonical Wnt signaling pathways [20-22]. Both pathways are critical for the proper development of the CNS. Functional studies have demonstrated that OFD1 has a crucial role in the formation of primary cilia, thus ascribing this pleiotropic disease to the growing number of disorders associated with dysfunction of primary cilia [20].

Previous studies aimed at defining the neuropathological aspects of OFD1 syndrome were based on a collection of cases in which a molecular diagnosis was not available [23-26]. Recent data reported both evaluation of brain MRIs from seven molecularly diagnosed OFD1 patients [9] and a role for Ofd1 in dorso-ventral patterning and axoneme elongation during embryonic brain development in the mouse [27]. Our larger cohort case study adds additional information towards a detailed characterization of the different types of CNS involvement observed in OFD1 patients.

## Materials and methods

### Collection of patients

A cohort of 117 female cases with a clinical and molecular diagnosis of OFD type 1 syndrome was assembled through an international effort. Most patients come from Europe (>65%) and North America (>19%), and the remaining from Australia, the Middle East and Asia. The majority of cases were Caucasians. Patients were assessed by clinicians at genetics centers worldwide and referred to the participating laboratories for mutation analysis of the *OFD1* gene. DNA or peripheral blood samples were accompanied by clinical data. The inclusion criteria were the presence of the Oral-facial-digital attributes characteristics of the OFD type 1 syndrome and, for the familial cases, the X-linked dominant-male lethal pattern of inheritance that is typical for OFD1.

The caring physicians evaluated this cohort of patients. On the basis of the available information supplied by the collaborating clinicians, we identified a selected cohort of seventy-one cases showing CNS involvement defined as any impairment of brain function (neurological symptoms, and/or cognitive/behavioral abnormalities) and/or structure (malformation/structural abnormalities) as evidenced on one hand by neurological examination, neuropsychological assessment, neurophysiological tests such as EEG and on the other by neuroimaging studies.

This selected cohort was further investigated through detailed neuroimaging studies as well as neurological examination. Neuropsychological testing by means of an ad hoc protocol was also proposed to the participating centers.

### Standard protocol approvals and patient consents

Written informed consent was obtained from all patients (or guardians) participating in the study. The study was approved by the French CPP (Comité de Protection des Personnes). Approval from an ethical committee was also obtained by the other universities and research institutes involved in this study.

### Mutation analysis

Mutation analysis was performed as described [7,8]. Negative cases were subjected to semiquantitative fluorescent multiplex method (QFMPSF), and relative quantification by real-time PCR (qPCR) to identify genomic rearrangements as described [28]. For all mutations 200 normal X-chromosomes from ethnically matched individuals were analyzed. Sequence variants were checked using the Mutalyzer program (http://www.LOVD.nl/mutalyzer) [29]. Nucleotide changes were verified and analyzed at http://genome.ucsc.edu/cgi-bin/hgGateway and at the single nucleotide polymorphism database at http://www.ncbi.nlm.nih.gov/projects/SNP/. Changes affecting splice-donor/acceptor-sites and flanking sequences were tested with the splice-site prediction software at http://www.fruitfly.org/seq_tools/splice.html.

### Neuroimaging

Magnetic Resonance Imaging (MRI) of the brain was performed following a standard protocol which included the following sequences: a) Turbo-Field-Echo (TFE) isometric 3-dimensional sagittal T1-weighted (T1-w) images with coronal and axial reconstructions and sagittal; b) axial and coronal Turbo-Spin-Echo (TSE) T2-weighted (T2-w) images; c) axial Fluid Attenuation Inversion Recovery (FLAIR) images and d) T1-w Inversion Recovery (IR) images.

Computed Tomography (CT) brain scans were all carried out without injection of contrast medium, with an average of 16 sections (4 mm thickness), using 100–150 mA in order to reduce the radiating dose.

Cranial ultrasonography (CUS) was performed using microconvex and multifrequency phase array transducers (5–7.5-10 MHz). The anterior fontanel was used as a window for standard coronal and sagittal planes, and in addition the whole brain was scanned to obtain a global view of the peri- and intraventricular areas and of the more superficial structures.

Post-mortem brain CT was performed with multislice spiral acquisition, while PM MRI included axial 3D ciss T2-w, 3D Flash T1-w, and Diffusion weighted (DW) sequences that were subsequently reconstructed on sagittal and coronal planes.

All supra- and infratentorial brain structures were evaluated on a qualitative basis, also for the lack of normal reference values in the paediatric age group.

Of note, it should be emphasized that the definition of CT is quite limited compared to MRI, particularly for the structures of the posterior fossa.

### Neurological and neuropsychological assessment

For each patient an accurate neurological examination protocol that included all the standard procedures was carried out.

The protocol proposed for developmental and neuropsychological assessment consisted of a battery of tests tailored to the patients’ age, cognitive level and degree of cooperation. Developmental assessment was carried out by means of the Griffiths Mental Development Scales (GMDS) for children aged 0–8 years, including six sub-scales: locomotor, personal-social, language, eye and hand coordination, performance and practical reasoning. Level of cognitive ability was tested with the Wechsler scales, namely the Wechsler Preschool and Primary Scale of Intelligence (WPPSI), the Wechsler Intelligence Scale for Children-IV (WISC) or the Wechsler Adult Intelligence Scale (WAIS), according to the [patient] child’s chronological age. In addition to the traditional Verbal IQ (VIQ), Performance IQ (PIQ), and Full Scale IQ (FSIQ) scores, four new indexes were introduced to study cognitive functions in further detail: the Verbal Comprehension Index (VCI), the Perceptual Organization Index (POI), the Freedom from Distractibility Index (FDI), and the Processing Speed Index (PSI) [30].

Intellectual disability was classified on the basis of the FSIQ score according to the Diagnostic and Statistic Manual of Mental Disorders (DSM-IV-TR): mild (50–69), moderate (35–49), severe (20–34), profound (below 20); a FSIQ between 70 and 84 indicated borderline intellectual functioning [31].

Motor and visual coordination was tested with the Developmental Test of Visual-Motor Integration (VMI) [32].

Visual/verbal learning and memory were tested with the Test of Memory and Learning (TOMAL) [33]. Visuospatial processing was assessed with the Rey-Osterreith Complex Figure test [34,35]. A subset of cases underwent a detailed assessment of language abilities according to the proposed neuropsychological protocol in which language tests were different according to mother tongues. In the three selected cases of whom we described in detail, the neuropsychological findings of Italians were analysed by means of the following tests: “Test del primo linguaggio” for children aged 18–36 months [36]; “TVL-Test di valutazione del linguaggio” [37] for children aged 3 to 6 years; Batteria per la valutazione dei disturbi del linguaggio in età evolutiva [38] for children aged 6 to 11.5 years.

Executive functions were evaluated with the Wisconsin Card Sorting Test, which measures the appropriateness of problem solving strategies in achieving a goal [39].

The behavioral and psychiatric assessment was performed with the Child Behavior Checklist (CBCL). The CBCL is a parent-report questionnaire with which the child can be rated on various behavioral and emotional problems. It assesses internalizing (i.e., anxious, depressive, and overcontrolled) and externalizing (i.e., aggressive, hyperactive, noncompliant, and undercontrolled) behaviors [40].

### Genotype-phenotype correlations

Possible genotype-phenotype correlation between each phenotypic neurological signs, brain structural abnormalities and cognitive defect, and type and position of mutations was investigated using contingency table analysis. The analyses of each phenotypic parameter were performed separately. The results were analysed by Fisher’s exact test to overcome the problem of small sample sizes.

## Results

### Mutational analysis

Mutations have previously been described for one hundred OFD1 patients [7,8,11,28]. Seventeen additional sporadic cases were identified.

For newly-identified cases, analysis of the coding region and introns flanking sequences of the OFD1 transcript was performed. Negative cases were then subjected to quantitative analysis to identify genomic rearrangements. These methods allowed the identification of 17 mutations reported in Table 1, which are scattered along the first 17 exons of the *OFD1* transcript that comprises 23 coding exons and encodes a 1011 Aa protein, as described [5]. Five out of the 17 point mutations and two genomic rearrangements were never reported before.

**Table 1:** Mutations identified in the present study*

***Exon/intron***	***Nucleotide change***	***Type of mutation***	***Predicted protein***	***Case***	***Mutation origin***	***Patient ID***
Ex 3	c.115C > T	Nonsense	p.Q39X	Sporadic	De novo	146°
	c.225C > G	Missense	p.N75K	Sporadic	De novo	145°
	c.275_276delCT	Frameshift	p.S92CfsX115	Sporadic	De novo	169
Ex5	c.400_403delGAAA	Frameshift	p.E134IfsX143	Sporadic	De novo	TH2
Ex 6	c.508_509delGA	Frameshift	p.D170FfsX173	Sporadic	NA	TH3
Ex 8	c.710dupA	Frameshift	p.Y238VfsX239	Sporadic	NA	148
	c.710dupA	Frameshift	p.Y238VfsX239	Sporadic	De novo	150
	c.710dupA	Frameshift	p.Y238VfsX239	Sporadic	NA	TH4
Ex 9	c. 914_915delAA	Frameshift	p.R306SfsX307	Sporadic	De novo	155°
Ex 11	c.1059 T > A	Nonsense	p.Y353X	Sporadic	De novo	159°
	c.1099C > T	Nonsense	p.R367X	Sporadic	De novo	156
	c.1099C > T	Nonsense	p.R367X	Sporadic	NA	147
	c.1128A > G	Splicing	-	Sporadic	De novo	151°
Int 11	c.1130-1G > A	Splicing	-	Sporadic	De novo	TH5
Ex 12	c.1193_1196del AATC	Frameshift	p.Q398LfsX400	Sporadic	De novo	161
**Genomic rearrangements:**						
Del ex 11	-	Genomic deletion	-	Sporadic	NA	139°
Del ex 7-10	-	Genomic deletion	-	Sporadic	NA	141°

*As reference, the A of the ATG translation initiation start site of the coding sequence for *OFD1* (Entrez nucleotide accession number NM_003611) is referred to as nucleotide +1.

NA, non-ascertained.

^o^not reported before.

### CNS involvement in OFD type 1

Based on available clinical information we selected all molecularly diagnosed OFD1 cases showing neurological symptoms and/or signs or cognitive/behavioral abnormalities (our selected cohort of seventy-one out of 117, 60,68%). The age ranged from 3 to 61 years, with the majority of patients being <18 years of age. Two cases were represented by aborted foetuses, and in these only neuropathological findings were available. The remaining patients were further investigated through a neuroimaging, neurological and neuropsychological protocol that was sent to all centers participating in the study. Assessment of younger children less than 4 years of age was performed using appropriate developmental scales such as the GMDS (see methods). These studies allowed for the identification of a wide spectrum of CNS involvement including structural abnormalities, neurological symptoms and signs and cognitive impairment. Additional file 1: Table S1 summarizes the genetic information and the clinical information (ethnicity, mail clinical features and nurological findings) for the entire cohort of patients.

#### Neuroimaging

**Figure 1:**
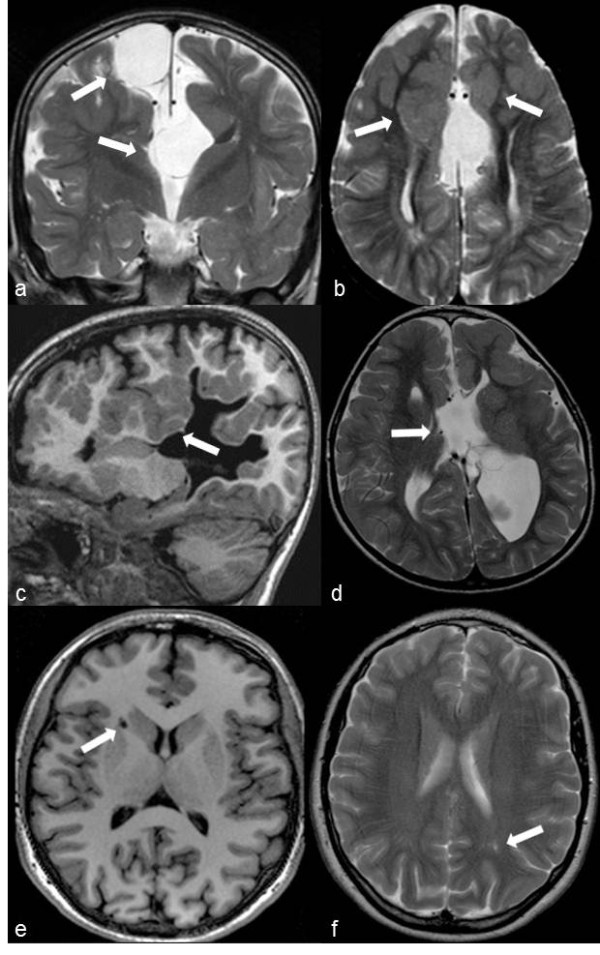
**Overview of the typical neuroimaging findings in ofd1 patients.** A, **b**: *Case ID50, 52 months* (*OFD1* mutation: 337 C > T; Q113X)*.***a)** Coronal TSE T2-weighted image: corpus callosum agenesis. Two arachnoid cysts (arrows) are respectively located upper to the roof of the third ventricle and on the right side of the falx. Note the “Texas longhorn” configuration well depicted only on the left side where the Probst bundle is also well visible. **b)** Axial TSE T2-weighted image: thinning and parallel orientation of the lateral ventricles. Bilateral fronto-mesial subcortical heterotopias (arrows) are evident along the anterior part of the interhemispheric defect. The bigger one anteriorly truncates the right Probst bundle that is thinner than the controlateral. A subtle thickening of the frontal cortical surfaces suggestive of polymicrogyria is also evident. Note the thin multiple dilatations of Virchow-Robin spaces at the posterior white matter. **c**, **d**: *Case ID39, 8-year-old* (*OFD1* mutation: 290 A > G; E97K)*.***c)** Sagittal TFE T1-weighted 3D images: note the frontal and parietal clefts surrounded by irregular and thickened grey matter consistent with infolding polymicrogyria (arrow). **d)** Axial TSE T2-weighted image: the interhemispheric defect is caused by the agenesis of the corpus callosum. Two arachnoid cysts are located between the outpouching of the third ventricle (arrow) and the enlarged left lateral ventricle. On the left side, polymicrogyria of the frontal cortical surface, as well as the periventricular deep portion of the infolding, are both well appreciable. **e**, **f**: *Case ID13, 13-year-old* (*OFD1* mutation: 247CAA > TAA; Q83X)*.***e**, **f)** Axial TFE T1weighted 3D and TSE T2 images: note some mild dilatation of Virchow-Robin spaces at the supratentorial white matter (arrows).

Among the dataset of patients displaying a neurological phenotype, forty-two cases (including two aborted fetuses) underwent MRI, two of these cases were also subjected to post mortem examination (namely a 7 year old girl who died of traumatic injury and one of the aborted fetuses), and 29 underwent brain CT scans or CUS as newborns. Eight patients among those subjected to CT scans had normal findings. Sixty-three cases (88.73%), including 42 patients subjected to MRI and 21 subjected to CT scans, showed one or more structural anomalies, namely: 45 cases displayed corpus callosum defects (agenesis or hypoplasia, ACC); arachnoid cysts were observed in 25 cases; 23 cases presented malformations of cortical development (MCD) (neuronal migration/organisation disorders, pachygyria, gray matter heterotopias, cortical dysplasia, polymicrogyria and schizencephaly); cerebellar developmental anomalies (CDAs) (cerebellar hypoplasia, isolated or associated with Dandy-Walker Malformation [DWM], or dysplasia) were described in 20 cases (DWM, in particular, was observed in 5 patients); cerebral atrophy and/or hypoplasia were detected in 9 cases; hydrocephalus or ventriculomegaly was observed in 8 cases; porencephaly was recognized in 3 cases, brain stem anomalies (hypoplastic and/or dysmorphic pons and/or medulla) in 2 cases, and hypothalamic hamartoma was found in one case. Table 2 summarizes these findings. Some patients displayed a more complex pattern of malformations (including agenesis of the corpus callosum, polymicrogyria, intracranial cysts, ponto-cerebellar hypoplasia and subependymal and subcortical heterotopia), while only mild anomalies could be detected in other cases, thus indicating a great degree of variability in the CNS involvement observed in these cases (Figure 1).

**Table 2:** Central Nervous System (CNS) structural abnormalities in our cohort of patients

***CNS structural abnormalities: type***	MRI/p.m.*	***eco/CT***	***Tot. N° (%)***^***a***^	***Tot. N° (%)***^***b***^
*Agenesis/hypoplasia of the Corpus Callosum (ACC)*	34/42 (80.95)	11/29 (37.93)	45/71 (63.38)	45/117(38.46)
*Intracerebral arachnoid cysts*	19/42 (45.24)	6/29 (20.69)	25/71 (35.21)	25/117(21.36)
*Malformations of cortical development (MCDs)*	22/42 (52.38)	1/29 (3.45)	23/71 (32.39)	23/117(19.65)
*Cerebellar developmental anomalies (CDAs)*	18/42 (42.86)	2/29 (6.9)	20/71 (28.17)	20/117(17.09)
*- Dandy-Walker Malformation (DWM)*	5/42 (11.90)	0/29 (0)	5/71 (7.04)	5/117(4.27)
*Cerebral atrophy/hypoplasia*	5/42 (11.90)	4/29 (13.79)	9/71 (12.68)	9/117(7.69)
*Hydrocephalus/ventriculomegaly*	6/42 (14.29)	2/29 (6.9)	8/71 (11.27)	8/117(6.83)
*Porencephaly*	1/42 (2.38)	2/29 (6.9)	3/71 (4.22)	3/117(2.56)
*Brain stem anomalies*	2/42 (4.76)	0/29 (0)	2/71 (2.82)	2/117(1.7)
*Hypothalamic hamartoma*	1/42 (2.38)	0/29 (0)	1/71 (1.41)	1/117(0.85)
***Total***	**42/42 (100)**	**21/29 (72.41)**	**63/71 (88.73)**	63/117(53.84)

*p.m. : post-mortem evaluation.

^a^percentages calculated on the total number of cases displaying CNS involvement.

^b^percentages calculated on the entire cohort of cases.

#### Neurological impairment

Sixty-nine out of the 71 patients showing CNS involvement were examined for signs and symptoms of neurological impairment. The two remaining cases were represented by aborted foetuses and were thus excluded from further evaluations. Table 3 describes the frequency of neurological disorders observed in patients with CNS structural anomalies (61 cases) compared to patients with normal neuroimaging findings (8 cases). Neurological impairment in patients with CNS structural abnormalities included motor delay (mild, moderate or severe) present in 15 cases, epilepsy - both generalized and focal seizures (infantile spasms, absences, tonic-clonic fits, drop attacks, focal motor seizures) – reported in 9 cases, hypotonia present in 5 cases (truncal in two cases and both truncal and limb in three cases), cranial nerve disorders in 5 cases (affecting, oculomotor and/or swallowing nerves), sensorineural hearing loss in 4 cases, spasticity of bilateral lower extremities in 3 cases, motor coordination deficits - both postural static and dynamic (gait ataxia ) – in 2 patients, hemiparesis in 2 cases and nystagmus in 2 cases. Patients without CNS structural abnormalities only showed either mild neurologic signs, such as motor coordination disorders (1 case) and/or mild motor delay (2 cases), or cognitive deficits (intellectual disability or selective disorders, see below). Epilepsy in one patient of the latter group may have the characteristics of a functional, not structural, disorder.

**Table 3:** Neurological disorders in our cohort of patients

***Neurological disorders: type***	***Patients with CNS structural abnormalities % (n°)***	***Patients without CNS structural abnormalities % (n°)***	***Total number of patients % (n°)***
*Motor delay*	24.6 (15/61)	25 (2/8)	24.64 (17/69)
*Epilepsy*	14.75 (9/61)	12.5 (1/8)	14.49 (10/69)
*Hypotonia*	8.2 (5/61)	0 (0/8)	7.25 (5/69)
*Cranial nerve disorders*	8.2 (5/61)	0 (0/8)	7.25 (5/69)
*Sensorineural hearing loss*	6.56 (4/61)	0 (0/8)	5.8 (4/69)
*Spasticity*	4.92 (3/61)	0 (0/8)	4.35 (3/69)
*Motor coordination deficits*	3.28 (2/61)	12.5 (1/8)	4.35 (3/69)
*Hemiparesis*	3.28 (2/61)	0 (0/8)	2.9 (2/69)
*Nystagmus*	3.28 (2/61)	0 (0/8)	2.9 (2/69)
***Total***	**55.74 (34/61)**	**37.5 (3/8)**	**53.62 (37/69)**

#### Neuropsychological assessment

A protocol for developmental and neuropsychological assessments of the 69 patients showing signs of CNS involvement (see above) was proposed to the participating centers. Twenty-two cases were reported to have normal cognitive levels on the basis of normal academic performances and/or normal adaptive functioning. Two cases were reported to have normal cognitive levels on the basis of specific neuropsychological testing. Forty-seven cases were referred as mentally impaired; for twenty-four of these cases, the assessment was based only on clinical observation, while for the other twenty-three patients, specific neurodevelopmental and/or neuropsychological testing was applied. In particular, four cases (ID50, ID13, ID39 and ID6) underwent a complete protocol assessing cognitive levels, and selective cognitive abilities (memory, learning and language). A complete description of three of these cases is reported below. The neuropsychological data collected revealed that, 40/69 cases (58%) showed a global intellectual disability (which were mostly mild, but moderate-severe deficits were also identified), and 7/69 cases (10%) demonstrated with selective cognitive impairment (defined on the basis of a normal full scale IQ) such as language disorders, learning difficulties or memory problems. Our results indicate that >68% of OFD1 cases from our selected cohort present some form of cognitive impairment. If we consider the entire cohort our data indicate that 40.8% of patients present some form of intellectual disability (47/115). Purely psychiatric disorders were quite rare: Attention Deficit Hyperactivity Disorder (ADHD) was found in two patients (one with and one without CNS anomalies) and Bipolar Disorder was observed in only one patient displaying CNS abnormalities.

Table 4 summarizes the cognitive/psychiatric defects observed both in patients displaying CNS structural anomalies and in those with normal neuroimaging findings. Isolated selective disorders were observed in 3/61 patients (4.92%) of the first group and in 4/8 (50%) patients of the second group. As expected moderate/severe intellectual disability was detected only in patients with CNS structural abnormalities (7/61, 11%).

**Table 4:** Cognitive/psychiatric defects in our cohort of patients

***Cognitive impairment: type***	***Cases with CNS structural abnormalities % (n°)***	***Cases without CNS structural abnormalities % (n°)***	***Total number of cases % (n°)***
*Mild MI/borderline intelligence:*	32.79 (20/61)	37.5 (3/8)	33.33 (23/69)
*Moderate/severe MI*	11.47 (7/61)	0 (0/8)	10.14 (7/69)
*MI (severity not specified):*	14.75 (9/61)	12.5 (1/8)	14.49 (10/69)
***Total***	**59.02 (36/61)**	**50 (4/8)**	**57.97 (40/69)**
*Selective cognitive impairment:*	4.92 (3/61)	50 (4/8)	10.14 (7/69)
*i) language disorders*	3.28 (2/61)	0 (0/8)	2.9 (2/69)
*ii) learning difficulties*	1.64 (1/61)	37.5 (3/8)	5.8 (4/69)
*iii) memory problems*	0 (0/61)	12.5 (1/8)	1.45 (1/69)
***Total (MI + selective cognitive impairment)***	**63.93 (39/61)**	**100 (8/8)**	**68.12 (47/69)**
*• Clinical assessment*	34.43 (21/61)	37.5 (3/8)	34.78 (24/69)
*• Specific testing*	29.51 (18/61)	62.5 (5/8)	33.33 (23/69)
***Psychiatric disorders: type***			
*ADHD*	1.59 (1/61)	12.5 (1/8)	2.9 (2/69)
*Bipolar disorders*	1.59 (1/61)	0 (0/8)	1.45 (1/69)
***Total***	**3.28 (2/61)**	**12.5 (1/8)**	**4.35 (3/69)**

MI: intellectual disability.

### Selected case reports

To illustrate the complexity and variability of the neurological phenotype observed in OFD1 we report the neuroimaging (MRI) and neuropsychological findings in three selected cases in detail.

#### Case ID50, 52 months (OFD1 mutation: 337 C > T; Q113X)

##### History and neurological examination

This young girl was born of non-consanguineous parents after an uneventful pregnancy and delivery; her birth weight was 3,150 grams. She was hospitalized soon after birth because of multiple congenital malformations (cleft lips and palate, scoliosis, right clubfoot, supernumerary lung lobe, trigonocephaly) and respiratory distress. Subsequently, she suffered from recurrent upper and lower airways infections. Neurological examination revealed a host of signs, which included a positive Romberg sign with broad-based staggering gait, dysmetria at the finger-to-nose test on the left side together with slight weakness of the left arm, brisk deep tendon reflexes with polyphasic left ankle jerk and right ankle clonus and positive right Babinski sign.

##### Neuroimaging

**Figure 2:**
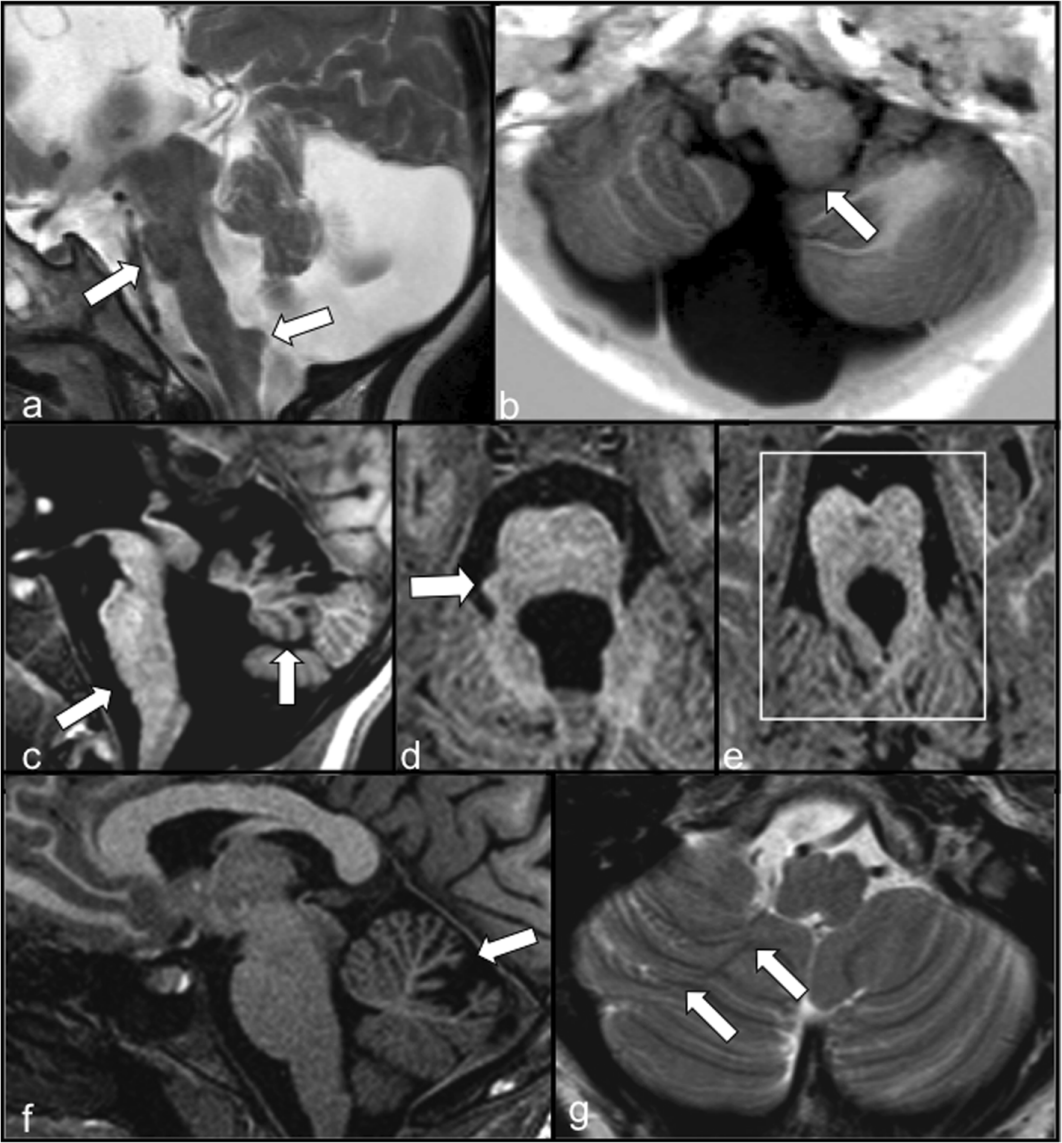
**Details of the neuroimaging findings from selected patients. a, b**: *Case ID50, 52 months* (*OFD1* mutation: 337 C > T; Q113X)*.***a)** Sagittal TSE T2-weighted image: a) vermis and pons hypoplasia (arrow) with anomalous elongation of the medulla, slight swelling of the tectum and aqueductal stenosis are shown together with an enlargement of the fourth ventricle and of the posterior fossa. An anomalous protuberance on the dorsal surface of the medulla is also evident (arrow). **b)** Axial IR T1-W image: dysmorphism of the left side of the medulla that is bigger than the controlateral one (arrow). Also note the hypoplasia of the cerebellar hemispheres with anomalous orientation of the folia on the right side. **c**, **d**, **e**: *Case ID39, 8-year-old* (*OFD1* mutation: 290 A > G; E97K)*.* c) Sagittal TFE T1weighted 3D image: marked hypogenesis, anticlockwise rotation (arrow) and dysmorphism of the vermis and flat ventral pons (arrow). The fourth ventricle and the posterior fossa are both enlarged. **d)** Axial TFE T1-weighted 3D image: little protuberance on the right side of the pons (arrow). e) Axial TFE T1 weighted image: typical “molar tooth sign” (square) characterized by thickening and horizontalization of the superior cerebellar peduncles. A mild hypointense dilatation of a Virchow-Robin space is present in the middle of the midbrain. The anomalous orientation of the cerebellar folia is also visible in the superior part of the cerebellum. **f**, **g**: *Case ID13, 13-year-old* (*OFD1* mutation: 247CAA > TAA; Q83X)*.***f)** Sagittal TFE T1-weighted 3D image: abnormal dilatation of the primary cerebellar fissure and hypoplasia of the declive lobule (arrow). **g)** Axial TSE T2-weighted image: enlargement of the right biventer lobule with abnormal orientation of the folia (arrows).

This case showed agenesis of the corpus callosum with two interemispheric arachnoid cysts (arrows in Figure 1a), bilaterally evident frontal polymicrogyria and fronto-mesial subcortical heterotopias (Figure 1b) and hypogenetic and dysplastic cerebellum (Figure 2a, b). The pons was clearly hypoplastic (Figure 2a), while the medulla was dysplastic due to abnormal hypertrophy on the left side (arrow in Figure 2b) with a protuberance on its dorsal surface (arrow in Figure 2a).

##### Neuropsychological assessment

Developmental assessment (Additional file 2: Table S2) by means of the GMDS showed a global developmental delay (mental age of 31.5 months) with worse performances in the locomotor domain. Language assessment revealed severe articulatory problems that made speech nearly incomprehensible, except for a few common words; language comprehension and grammar were quite appropriate for the developmental level. Behavioural assessment by means of the CBCL detected problems in the attention domain (Additional file 3: Table S3).

#### Case ID39, 8-year-old (OFD1 mutation: 290 A > G; E97K)

##### History and neurological examination

This girl was born following a caesarean section at the 29^th^ week of gestation after an abnormal pregnancy with oligohydramnios and inadequate intrauterine growth. Her birth weight was 990 grams. She was transferred to the neonatal intensive care unit due to respiratory distress and intracranial haemorrhage. Multiple congenital anomalies were also noted: cleft tongue, oral aberrant frenula, syndactyly, facial dysmorphisms (hypotelorism and anteverted nostrils), interatrial defect (ostium secundum), corpus callosum agenesis, right kidney hypoplasia, and presence of bilateral renal cysts. Subsequently, she suffered from febrile seizures and also from afebrile fits that were not considered to be of epileptic origin. Upon neurological examination, the girl showed a clumsy gait with difficulty walking on tiptoes. Moreover, clumsiness was also evident in her spontaneous motor activities. Muscle tone was normal as well as deep tendon reflexes.

##### Neuroimaging

The corpus callosum was absent with two interhemispheric arachnoid cysts (Figure 1d). Frontal and parietal polymicrogyria were evident (arrows in Figure 1c,d).

The cerebellum was hypogenetic and dysplastic, showing an upper anticlockwise rotation of the vermis (arrow in Figure 2c). A typical “molar tooth” sign was evident (square in Figure 2e). The posterior fossa and the fourth ventricle were both enlarged (Figure 2c), and the ventral pons was flat with a protuberance on its right side (arrows in Figures 2c,2d).

##### Neuropsychological assessment

Developmental assessment (Additional file 2: Table S2) evaluated with the GMDS showed a global developmental delay in the moderate-severe range (mental age of 35 months). The best performance was in the “practical reasoning” domain, while the locomotor subscale gave the lowest score. Behavioural assessment by means of the CBCL revealed clear-cut attention problems (Additional file 3: Table S3). On the whole, the cognitive profile was similar to the one described for the previous patient.

#### Case ID13, 13-year-old (OFD1 mutation: 247CAA > TAA; Q83X)

##### History and neurological examination

This girl was born at term weighing 3,450 grams after a pregnancy complicated by hyperemesis and recurrent abdominal pain. A diagnosis of OFD1 syndrome was entertained in the neonatal period due to the presence of cleft tongue, supernumerary gingival frenula, nasal and forehead milia, and nuchal alopecia. Polycystic kidney disease, as well as a mitral valve prolapse, was subsequently detected with no remarkable clinical consequences. Neurological examination was normal.

##### Neuroimaging

The brain structure was normal except for a mild dilatation of some Virchow-Robin spaces (arrows in Figure 1e,f). The cerebellum showed an abnormal dilatation of the primary cerebellar fissure with hypoplasia of the declive lobule (arrow in Figure 2f) and an enlargement of the right hemispheric biventer lobule (arrows in Figure 2g).

##### Neuropsychological assessment

Global intellectual functioning at the WISC-III scale was at the lowest normal score; more precisely, the Verbal Comprehension Index was very low, while the Perceptual Organization Index as well as Freedom from Distractibility Index and Processing Speed Index, was in the normal range. Executive functions assessed by the “Wisconsin Card Sorting Test” were impaired by a high number of perseverant answers. Verbal fluency was poor with associated semantic-lexical difficulties and problems in recalling words or names (lexical deficit of the anomia type). Long-term verbal memory was borderline while both overall non-verbal memory and short-term verbal memory were within the normal range. Details of the aforementioned neuropsychological assessment are given in Additional file 4: Table S4).

Behavioral assessment by means of the CBCL revealed a withdrawn profile pointing to reduced social intercourse and introversion (Additional file 3: Table S3).

##### Genotype/phenotype correlation

Statistical analysis was performed but no significant correlations could be identified between the site and extent of CNS abnormalities, the cognitive impairment and the type and position of mutations.

## Discussion

We report a detailed characterization of the CNS involvement observed in a large cohort of OFD type 1 cases. In our study the CNS involvement is defined as the presence of neurological symptoms, and/or cognitive/behavioral abnormalities and/or brain structural abnormalities. Our study, based uniquely on patients with clearly defined pathogenic mutations, indicates that over 60% of the full cohort of 117 cases displays a CNS involvement. These results are in agreement with similar findings in the literature [7,9,41]. We also compared results from this study to those described in Bisschoff et al. (Table 5). Our results indicate that, in our full cohort, 63 out of 117 cases (53.8%) display brain malformations/structural abnormalities. This percentage rises up to 88.7% in our selected cohort of 71 cases subjected to neuroimaging studies and could further increase if modern brain neuroimaging techniques would be carried out also in asymptomatic/mildly-affected patients. Our results also indicate that some form of intellectual disability ranging from mild to severe and selective cognitive impairment can be detected in >68% of cases in our selected cohort.

**Table 5:** CNS involvement in OFD type I

***CNS involvement in OFD1***	***Bisshoff et al. ***[9]	***This report***	***Tot. N° (%)***
*Total CNS involvement*	20/31 (64.5)	71/117 (60.68)	91/148 (61.49)
*Available MRI*	7/31 (22.5)	42/71 (59.15)	49/102 (41.18)
*Total CNS malformations*	NR	63/71 (88.73)*	63/71 (88.73)
*Agenesis/dysgenesis Corpus Callosum*	13/16 (81.2)	34/42 (80.95)^a^	47/58 (81.03)
*Malformations cortical development (MCDs)*	4/6 (66.6)	22/42 (52.38)^a^	26/48 (54.17)
*Cysts*	7/13 (53.8)	19/42 (45.24)^a^	26/55 (47.27)
*Hydrocephalus/Porencephaly*	8/13 (61.5)	7/42 (16.66)^a^	15/55 (27.27)
*Cerebellar developmental anomalies (CDAs)*	NR	18/42 (42.86)^a^	18/42 (42.86)
*Cerebral atrophy/hypoplasia*	NR	5/42 (11.90)^a^	5/42 (11.90)
*Brain stem anomalies*	NR	2/42 (4.76)^a^	2/42 (4.76)
*Hypothalamic hamartoma*	NR	1/42 (2.38)^a^	1/42 (2.38)
*MI/ psychomotor retardation*	12/26 (46.1)	47/69 (68.12)	59/95 (62.1)
*Epilepsy*	4/25 (16.0)	10/69 (14.49)	14/94 (14.89)

NR: not reported.

MI: intellectual disability.

*based on MRI/p.m and eco/CT evaluations.

^a^based only on MRI/p.m findings.

Statistical analysis failed to show a straightforward genotype–phenotype correlation; however, the number of patients analyzed and the possible importance of environmental factors warrant further investigation.

Our study allowed us to determine the incidence of the different types of CNS structural abnormalities. In particular ACC, MCDs, CDAs, and intracerebral cysts were the most frequently observed malformations. However, it is important to underscore that given the quite limited definition of CT compared to MRI, particularly for the structures of the posterior fossa, a percentage of missed structural brain anomalies (related to the cases who did not undergo MRI studies) should be taken into account.

Taken together, all the above mentioned malformations might be related to disorders of cellular migration and proliferation in the developing brain.

We questioned whether the presence of structural brain anomalies could be significantly associated with cognitive and/or neurological impairment. Only intellectual disability as a whole (mild, moderate or severe) appeared to be more frequently associated with brain structural anomalies, while selective neuropsychological defects (language disorders, learning disorders, memory deficits) behaved independently. As expected, a higher prevalence of neurologic signs was found in patients with brain malformations. MCDs and CDAs could represent a possible neurobiological basis for the cognitive impairment observed in OFD type 1 syndrome. This observation is based on data demonstrating that neuronal migration defects result in neurological impairment, including in intractable epilepsy and intellectual disability [42], and also on the accumulated evidence on the role of the cerebellum in the context of child development and learning processes [43].

Mutations in the OFD1 gene have also been reported in an X-linked recessive intellectual disability syndrome comprising macrocephaly and ciliary dysfunction [12], Joubert (JBTS10) syndrome [13-16] and in X-linked retinitis pigmentosa [17].

Joubert syndrome and related disorders (JSRD) spectrum is primarily defined by the presence of a specific neuroimaging hallmark- the “molar tooth sign” (MTS)-resulting from a specific midbrain-hindbrain malformation (thickened, elongated and horizontally located superior cerebellar peduncles together with an abnormally deep interpeduncular fossa and vermian hypo-dysplasia) [44]. Additional infratentorial and supratentorial neuroimaging findings reported in JSRDs include, for the first set enlargement of the posterior fossa and fourth ventricle, reduced or enlarged size of the cerebellar hemispheres and abnormal brain stem morphology. Supratentorial findings include migration disorders, callosal dysgenesis, ventriculomegaly and hippocampal malrotation. Encephaloceles, most often located in the occipital regions, have also been described. The cognitive phenotype is characterized by delayed language and motor skills. Mild to severe intellectual disability is often described, but exceptional cases may have borderline or even normal intelligence [45].

MTS should also be considered the main diagnostic criterion for OFDVI [46]. Interestingly, two OFDVI patients were reported to have a homozygous mutation in *TMEM216*, a gene implicated in JSRD and Meckel-Gruber syndrome (MKS) [47], and a mutation in the *OFD1* transcript has been reported in an OFDVI case [48]. More recently a high frequency of mutations in C5ORF42 has been reported in OFDVI patients [49]. One out of the 71 OFD1 cases that we have analyzed displayed a typical MTS. The neurological features observed in OFD type 1 and JSRDs suggest that OFD1 can be considered an extension of the JSRD spectrum. OFD1, OFDVI and JSRDs could represent different levels of expression of the ciliary dysfunction in the brain.

The spectrum of CNS malformations in MKS ranges from total craniorachischisis to a partial agenesis of the corpus callosum. Neuropathological studies have shown prosencephalic dysgenesis, defects in midline formation, polymicrogyria, heterotopias, and neuroepithelial rosettes [50]. Neurodevelopmental defects in the form of more subtle brain tissue- and region-specific abnormalities are frequently reported in Bardet-Biedl (BBS) patients [51]. As for the cognitive phenotype, a characteristic profile including low IQ, impaired fine motor skills, and decreased olfaction has been identified in BBS patients [52].

Our findings well correlate with the brain developmental anomalies described in ciliopathies. In addition, we describe the presence of characteristic features that have not been previously reported in OFD1 proven cases. We documented cerebellar developmental anomalies including rotation of the vermis, as well as segmental anomalies such as hypoplasia of the declive lobule or enlargement of the right biventer lobule and abnormal orientation of hemispheric folia and sulci. More interestingly, for the first time we report a peculiar dysplastic feature of the brainstem, characterized by the presence of abnormal protuberances. Jurich-Sekhar et al. recently described excess tissue in the brainstem of two JSRDs cases bearing mutations in *OFD1*[15]. However, the two patients did not display the obligatory hallmark of JSRDs, the MTS. This dysplastic abnormality may also reflect disturbances of patterning and cell migration related to neurodevelopmental functions of primary cilia.

Consistent with various neurological symptoms detected in ciliopathic patients, most cells in the brain (including neural progenitors and mature neurons, glial cells/astrocytes and ependymal cells) have primary cilia [53]. These organelles have a definite role in brain development: brain patterning is controlled by morphogens such as Shh, Wnt and Fgf, which require primary cilia to effectively transduce their signals [2,54]. Defective ciliary function resulting in impaired signal transduction might explain the severe malformations occurring in the early stages of brain development. In addition, ciliary proteins have been implicated in control of centrosome/centriole positioning possibly leading to abnormal neuronal migration early in development [55]. Interestingly cilia have also been shown to orchestrate the coordinated migration and placement of postmitotic interneurons in the developing cerebral cortex [56,57], Moreover OFD1 has been shown to play a role in the neuronal differentiation of embryonic stem cells [58].

Cognitive impairment in OFD1 syndrome also questions the role of primary cilia in more advanced brain function. Cilia are critical regulators of Shh signaling on postnatal precursor cells and participate in orchestrating postnatal forebrain development and stem/precursor cell maintenance [59]. Impaired Shh signaling due to cilia dysfuction leads to defective hippocampal morphogenesis and dysregulation of mitotic activity in the mice’s postnatal brain [59], stressing the hippocampus as a target organ for CNS-related ciliopathic manifestations.

Interestingly, as far as neuropsychological aspects are concerned, some common traits can be isolated, namely a reduced ability in processing verbal information, a slow thought process, difficulties in attention and concentration, and notably, long-term memory deficits. Similar neuropsychological features associated with high percentage of hippocampal dysgenesis were reported in a cohort of patients with BBS syndrome [60]. Moreover, in Joubert syndrome, Poretti et al. [61] reports on difficulties in some executive functions as we found in our patient ID13. In the subset of patients for whom a detailed neuropsychological assessment was available no significant hippocampal abnormalities have been detected. Recent data obtained in mouse models show that hippocampal neurogenesis, in particular in the adult mouse, requires intact primary cilia. It has been reported that the neuronal primary cilia of the hippocampal dentate gyrus are the drivers of neurogenesis and memory formation [62]. It would be beneficial to evaluate how deficient ciliary function affects learning and memory in patients, as adult hippocampal neurogenesis has a crucial role in memory, and newborn neurons of the dentate gyrus might be involved in pattern integration and pattern separation [63]. Evidence for involvement of cilia in higher brain functions is now available. Loss-of-function of the somatostatin receptor 3 (SSTR3), localized to cilia in the neocortex and hippocampus leads to impaired object recognition in mice, whereas the loss of other SSTRs, not found on cilia, does not [64]. This only happens in mature neurons inasmuch as SSTR3 is only evident in the brain post-natally [65].

## Conclusions

In conclusion our data support the finding that over 60% of OFD1 patients show neurodevelopmental defects including brain structural anomalies, cognitive impairment (borderline intelligence, mild to severe intellectual disability and selective cognitive defects), and associated neurological disorders and signs. Our data also revealed that cognitive impairment is observed in over 40% of cases and this percentage increases up to 88% in our selected cohort. On the basis of our results a full neurological, neuroimaging and neuropsychological evaluation is recommended to allow a better management of OFD type 1 patients. Our results expand our knowledge of CNS involvement in ciliopathies. Conditional inactivation of the *Ofd1* transcript in the murine hippocampus at different developmental stages and in adult brains will help in clarifying the role of Ofd1 and primary cilia in higher brain functions.

## Abbreviations

ACC: Agenesis of the corpus callosum; ADHD: Attention deficit hyperactivity disorder; BBS: Bardet-Biedl syndrome; CBCL: Child behavior checklist; CDA: Cerebellar developmental anomalies; CNS: Central nervous system; CT: Computed tomography; DWM: Dandy-Walker Malformation; DSM-IV-TR: Diagnostic and statistic manual of mental disorders; FDI: Freedom from distractibility index; FSIQ: Full scale IQ; GMDS: Griffiths mental development scales; JBTS: Joubert syndrome; JSRD: Joubert syndrome related disorders; MCD: Malformation of cortical development; MKS: Meckel-Gruber syndrome; NMDs: Neuronal migration disorders; OFD: Oral-facial-digital; PIQ: Performance IQ; POI: Perceptual Organization Index; PSI: Processing Speed Index; QFMPSF: Semiquantitative fluorescent multiplex method; qPCR: Relative quantification by real-time PCR; VIQ: Verbal IQ; VCI: Verbal Comprehension Index; VMI: Visual-Motor-Integration; WPPSI: Wechsler Preschool and primary scale of intelligence; WISC: Wechsler intelligence scale for children-IV; WAIS: Wechsler adult intelligence scale.

## Competing interest

The authors declare that they have no competing interests.

## Authors’ contribution

EDG conceived the study, participated in its design and coordination and helped to draft the manuscript. MM carried out the molecular genetic studies and drafted the manuscript. FI carried out the neuropsychological studies. ADA performed neuroimaging studies for Italian patients. PP, LP, VL, SL, VS-D performed detailed analysis of clinical data. CTR participated in the design and coordination of the study. BF conceived the study and participated in its design and coordination and helped to draft the manuscript. All authors read and approved the final manuscript. Members of the Oral-Facial-Digital Type I (OFD1) Collaborative Group referred cases and performed analyses of clinical data on referred cases.

## Supplementary Material

Additional file 1: Table S1 Genetic and clinical information of the cohort of OFD1 patients. Click here for file

Additional file 2: Table S2 Details of developmental assessment in patients ID50 and ID39. Click here for file

Additional file 3: Table S3 Details of behavioral assessment in patients ID50, ID39 and ID13. Click here for file

Additional file 4: Table S4 Details of neuropsychological assessment in patient ID13. Click here for file

## Members of the OFD1 collaborative group

Australia: A Bankier/S White (Royal Children’s Hospital Genetics Clinic, Melbourne), F Collins (The Children’s Hospital at Westmead, Sidney), M Gardner (Royal Children’s Hospital, Victoria), SL Keeling, T Tan (Royal Children’s Hospital Genetics Clinic, Victoria), J McGaughran/F McKenzie (Royal Children’s Hospital, Herston); Austria: K Lhotta (University Hospital, Innsbruck); Bahrain: F Abdulla (Salmaniya Medical Center, Riyadh); Belgium: A Destree (Institut de Pathologie et de Génétique, Gosselies), K Devriendt/G Matthijs (Center for Human Genetics, Leuven); Canada: R Ferrier (University of Calgary), DR McLeod (Alberta Children’s Hospital, Calgary), JM Friedman (British Columbia’s Children’s Hospital, Vancouver), H Heran (Children’s and Women’s Health Center of British Columbia, Vancouver), GE Graham (Children’s Hospital of Eastern Ontario, Ottawa), R Klatt/A Teebi (The Hospital for Sick Children, Toronto); Denmark: P Jensen (University Hospital, Aarhus); France: B Gilbert (Université de Poitiers, Poitiers), S Marlin (Hospital D’Enfants Armand Trousseau, Paris), A Toutain (Bretonneau University Hospital, Tours), A David (Service de Génétique Médicale, Nantes), S Odent (Service de Génétique Clinique, Rennes), D Héron (Unité Fonctionnelle de Génétique Médicale, Groupe Hospitalier Pitié Salpêtrière, Paris), L Burglen (Hôpital Trousseau, Trousseau), M Rio (Hôpital Necker-Enfants Malades), PS Jouk (Centre Hospitalier Universitaire, Grenoble), G Plessis (Service de Génétique, CHRU, Caen), J Lespinasse (Génétique Médicale, Chambéry), F Giuliano/C Turc-Carel (University Hospital Nice); Germany: RC Betz (Rheinische Friedrich-Wilhelms-Universitat, Bonn), S Heim (Aachen University, Aachen), M Klehr-Martinelli (Prenatal Medicine Molecular Genetics Laboratory, Munich), D Kotzot (University of Munich), M Minnerop (Research Centre Juelich, Institute of Neuroscience and Medicine, Jülich), C Schell-Apacik (Children’s Center, Munich), A Gal/U Orth (University Hospital Eppendorf, Hamburg), G Gillessen-Kaesbach (Universitatsklinikum, Essen), B Zoll (Georg-August University, Goettingen), J Mucke/A Tzschach (Children’s Hospital, St. Ingbert), E Godde (Witten/Herdecke University, Datteln); Israel: R Carmi (Ben-Gurion University of the Negev, Beer-Sheva); Italy: N Brunetti/A Scarcella (Federico II University, Naples), P Castelluccio (Medical Genetics Unit, A. Cardarelli Hospital, Naples), C Castellan (Azienda Sanitaria, Bolzano), O Gerola (Policlinico S. Matteo, Pavia), S Bigoni (Arcispedale Sant’Anna, Ferrara), L Zelante (Casa Sollievo della Sofferenza, S. Giovanni Rotondo Foggia), A Sabato (Borgo Roma Hospital, Verona); G Bianchini/L Garavelli (Arcispedale S. Maria Nuova, Reggio Emilia), R Virdis (Clinica Pediatrica Università di Parma, Parma), GB Ferrero (Clinical Genetics Unit, University of Turin, Turin), A Selicorni (Clinica Pediatrica De Marchi, University of Milan), F Gurrieri (University Cattolica S.Cuore, Rome); Lebanon: A Megarbane (Saint-Loseph University, Beirut); Philippines: MA Chiong/EM Cutiongco (Institute of Human Genetics, University of the Philippines, Manila); Poland: E Obersztyn, A Kutkowska-Kazmierczak (National Institute of Mother and Child, Warsaw); Portugal: CR Mota (Institute of Medical Genetics D. Jacinto de Magalhaes, Porto); Serbia: G Stevanovic (Clinic for Neurology and Psychiatry for Children and Youth, Belgrade); Spain: J Sanchez Del Pozo (University Hospital, Madrid), MG Barcina (Hospital of Basurto, Bilbao); Sweden: E Iwarsson (Karolinska University Hospital, Stockholm); Switzerland: V Graber, R Okhowat/A Shinzel (University of Zurich, Zurich); the Netherlands: HG Brunner (University Hospital, Nijmegen), I Krapels/V Hovers (University Hospital, Maastricht), FA Beemer/P Terhal (University Medical Center, Utrecht), P Rump (University Medical Centre, Groningen); Turkey: N Elcioglu (Marmara University Hospital, Istanbul), O Toprak (Ataturk Research and Training Hospital, Izmir); UK: J Burn, A Henderson/E Jones (Institute of Human Genetics, International Centre for Life, Newcastle upon Tyne), J Dean (Department of Medical Genetics, NHS Grampian, Aberdeen), B Castle (University Hospital Trust, Southampton), F Macdonald, P Farndon, D Williams (Birmingham Women’s Hospital, Birmingham), T Homfray/R Taylor (St. George’s Hospital Medical School, London), M Lees/S Loughlin (Great Ormond Street Hospital for Children NHS Trust, London), FL Raymond, D Trump, J Whittaker (Addenbrooke’s Hospital, Cambridge), S Smithson (St. Michael’s Hospital, Bristol), J Rankin, C Turner (Royal Devon and Exeter NHS Trust, Exeter); USA: L Bird/J Chibuk/D Masser-Frye (University of California, San Diego), S Sell (Medical Center, Hershey), S Amy/I Schafer (Cleveland Clinic Foundation, Cleveland), LE Bartoshesky/K Jenny (A. duPont Hospital for Children, Wilmington), P Benke (Hollywood Memorial Hospital, Florida), C Curry/A Swenerton/T Treisman (Children’s Hospital Central California, Madera), JW Dunlap/V Shashi (Wake Forest University School of Medicine, North Carolina), E Reich (New York University School of Medicine, New York), T Reimschisel (Washington University, St Louis), R Pfau (The Children’s Medical Center, Dayton), B Pober (Yale University, New Haven), J Robertson (Henry Ford Hospital, Detroit), J Roggenbuck (Children’s Hospital of Minnesota, Minneapolis), H Thiese (Group Health Cooperative, Seattle).
