# Supplementary material for: CNS involvement in OFD1 syndrome: a clinical, molecular, and neuroimaging study
Source: Orphanet J Rare Dis. 2014 May 10;9:74. doi: 10.1186/1750-1172-9-74 (PMC4113190; doi:10.1186/1750-1172-9-74)
Supplement: Additional file 1: Table S1 — Genetic and clinical information of the cohort of OFD1 patients. [file 1750-1172-9-74-S1.docx]

| Additional file 1: Table S1. Genetic and clinical information of the cohort of OFD1 patients | | | | | | | | | | | | | | |
| --- | --- | --- | --- | --- | --- | --- | --- | --- | --- | --- | --- | --- | --- | --- |
| ID | MUTATION | SITE | TYPE | CASE | AGE | ORIGIN | CRANIO-FACIAL | ORAL | SKELETAL | KIDNEY | RMN | TC/ECO | NEUROLOGICAL | COGNITIVE and  PSYCHIATRIC |
| 81 | c.65dupA | ex 2 | fr | sporadic | 12y | EU | dysm  lip | tongue  fren  cleft  teeth | limbs | - | - | ACC | N | mMI |
| 100 | c.111G>A | ex 2 | splice | sporadic | 30y | EU | N | tongue  teeth | N | - | - | - | - | - |
| 101 | c.111G>C | ex 2 | splice | sporadic | 38y | EU | milia | tongue  palate  teeth | limbs | PKD | ACC  MCDs | - | N | LeD |
| 134 | c.43_44delAG | ex 2 | fr | sporadic | 4y | EU | dysm | fren | limbs | - | - | Cy | N | N |
| 53 | c.111+2T>C | int 2 | splice | sporadic | 6y | NA | dysm | tongue  palate | limbs | - | - | ACC  MCDs  CDAs | cnD | N |
| 3 | c.294_312del TGGTTTGGCAAAAGAAAAG | ex 3 | fr | familial | 36y | EU | hair  dysm  lip | palate | limbs | PKD | - | CA | N | mMI |
| 10 | c.121C>T | ex 3 | nons | familial | 4y | EU | hair  dysm | fren | limbs | - | - | - | - | - |
| 13 | c.247C>T | ex 3 | nons | sporadic | 16y | EU | hair | tongue  fren  teeth | limbs | PKD | CDAs | - | N | Bi |
| 17 | c.221C>T | ex 3 | miss | sporadic | 19y | EU | dysm | tongue  fren  palate  teeth | limbs  short | - | MCDs  CDAs | - | E | LaD |
| 28 | c.243C>G | ex 3 | miss | sporadic | 12y | EU | milia  dysm | tongue  palate | limbs | PKD | MCDs  CDAs  ACC | - | MoCo  E | mMI |
| 38 | c.313dupG | ex 3 | fr | familial | 39y | EU | milia  dysm  lip | tongue  palate | limbs | - | - | Cy | N | N |
| 38a | c.313dupG | ex 3 | fr | familial | 17y | EU | Lip | tongue  fren | N | PKD | - | Por | N | N |
| 39 | c.290A>G | ex 3 | miss | familial | 8y | EU | dysm | tongue  fren | limbs | - | MCDs  CDAs  ACC | - | N | moMI |
| 74a | c.241C>G | ex 3 | miss | sporadic | 15y | EU | N | tongue fren  palate  teeth | limbs | - | ACC  CDAs | - | Hy | mMI |
| 74b | c.241C>G | ex 3 | miss | sporadic | 15y | EU | N | tongue  fren  palate  teeth | limbs | - | ACC  Cy  CDAs  (DWM) | - | Hy  S | moMI |
| 83 | c.260A>G | ex 3 | miss | familial | 6y | NA | N | tongue  cleft  palate  teeth | limbs | - | CDAs  (DWM)  ACC  Cy  MCDs | - | cnD | N |
| 94 | c.224A>C | ex 3 | miss | sporadic | 5y | EU | hair  dysm | tongue  fren  palate | limbs | - | - | ACC | N | N |
| 111 | c.274T>C | ex 3 | miss | sporadic | 45y | EU | milia  dysm | tongue  palate  teeth | limbs | PKD | - | - | - | - |
| 121 | c.162_166delTGGAG | ex 3 | fr | familial | 5y | NA | milia  hair  dysm | tongue  fren | limbs | PKD | - | Cy | N | N |
| *145 | c.225C>G | ex 3 | miss | sporadic | 4y | EU | hair  dysm | tongue  palate | limbs | - | - | CA | N | N |
| *146 | c.115C>T | ex 3 | nons | sporadic | 3y | EU | milia  hair  dysm | tongue  fren  cleft | limbs | - | - | - | - | - |
| *169 | c.275_276delCT | ex 3 | fr | sporadic | 4y | EU | milia  hair  dysm | tongue  fren  teeth | limb | - | H  Cy  ACC  MCDs | - | cnD | mMI |
| 50 | c.337C>T | ex 4 | nons | familial | 7y | EU | dysm | tongue  fren  palate | limbs | - | MCDs  ACC  Cy  BrA  CDAs  (DWM) | - | He  S  MD  Ny  MoCo | mMI |
| 65 | c.372C>G | ex 4 | nons | sporadic | 8y | NA | milia  dysm | tongue  fren  cleft | limbs | - | ACC  MCDs | - | N | mMI |
| S6 | c.412G>A | ex 4 | miss | sporadic | 16y | EU | lip  dysm | tongue  fren  palate  teeth | limb | - | ACC | - | MD | mMI  ADHD |
| 64 | c.382-3C>G | int 4 | splice | sporadic | 8y | EU | dysm | tongue  fren | limbs | PKD | - | ACC | N | N |
| 133 | c.382-2A>G | int 4 | splice | sporadic | 19y | EU | hair | tongue  fren  palate | limbs | PKD | - | - | - | - |
| 14 | c.400_403delGAAA | ex 5 | fr | sporadic | 22y | EU | hair  dysm | tongue  fren  teeth | limbs | - | - | N | N | LeD |
| 117 | c.411delA | ex 5 | fr | sporadic | 12y | EU | N | tongue  palate  teeth | limbs | - | H  MCDs | - | MD | MI |
| 118 | c.400_403delGAAA | ex 5 | fr | sporadic | 57y | EU | hair  dysm  lip | tongue  palate | limbs  short | PKD | - | - | - | - |
| *TH1 | c.400_403delGAAA | ex 5 | fr | sporadic | 4y3m | EU | dysm  lip | tongue  fren  palate | limb | - | ACC  CDAs  MCDs | - | MD | sMI |
| *TH2 | c.400_403delGAAA | ex 5 | fr | de novo | fetus | EU | dysm | palate  tongue  fren | limb | - | CA  Cy  ACC  CDAs  MCDs  BrA | - | N | N |
| 15 | c.412+2delT | int 5 | splice | sporadic | 9y | EU | Lip | tongue  fren  palate | limbs | - | Cy  ACC  H  CDAs  MCDs | - | Hy  Ny  MD | sMI |
| 16 | c.431dupT | ex 6 | fr | sporadic | 32y | EU | dysm  lip | tongue  fren  palate | limbs | PKD | - | - | - | - |
| 68 | c.454C>T | ex 6 | nons | sporadic | 28y | EU | dysm | tongue  palate | limbs | - | - | - | - | - |
| S5 | c.431T>A | ex 6 | nons | sporadic | 17y | EU | dysm  hair | tongue  fren  teeth | limb | PKD | ACC  H  Cy | - | E  S  MD | mMI |
| *TH3 | c.508_509delGA | ex 6 | fr | sporadic | 23y | EU | dysm  lip | tongue  fren  teeth | N | - | MCDs  ACC  Cy | - | N | N |
| 69 | c.602delA | ex 7 | fr | sporadic | 12y | EU | dysm | tongue  fren  teeth | limbs | - | - | - | - | - |
| 79 | c.616_617delGA | ex 7 | fr | sporadic | 7y | NA | milia  hair | tongue  fren  cleft  teeth | limbs | - | Cy  MCDs  H | - | N | N |
| 88 | c.653delA | ex 7 | fr | familial | 11y | EU | hair  dysm | tongue | limbs | - | - | - | - | - |
| 113 | c.594_598delAAAGC | ex 7 | fr | familial | 28y | NA | milia | tongue  fren  palate  teeth | limbs | PKD | - | - | - | - |
| 135 | c.628C>T | ex 7 | nons | sporadic | 23y | NA | dysm | tongue  fren  teeth | limbs | - | - | - | - | - |
| 61 | c.654+2_654+4delTA | int 7 | splice | sporadic | 7y | NA | milia  hair  dysm | tongue  fren  cleft  palate | limbs | - | - | - | - | - |
| 7 | c.710dupA | ex 8 | fr | familial | 20y | EU | milia | N | limbs | PKD | - | - | - | - |
| 8 | c.710dupA | ex 8 | fr | familial | 32y | EU | hair  dysm | fren  teeth | limbs  short | PKD | - | - | - | - |
| 21 | c.710dupA | ex 8 | fr | familial | 12y | EU | milia | tongue  fren  palate | limbs | - | - | Cy | N | N |
| 32 | c.710delA | ex 8 | fr | sporadic | 14y | NA | milia | tongue  fren  teeth | limbs | - | - | - | - | - |
| 34 | c.710dupA | ex 8 | fr | sporadic | 9y | NA | milia  lip | tongue  fren  palate | limbs | - | - | - | - | - |
| 46 | c.823C>T | ex 8 | nons | sporadic | 8y | EU | milia  dysm | tongue  fren  palate | limbs | PKD | - | ACC  Cy | Hy  MD | sMI |
| 70 | c.710dupA | ex 8 | fr | familial | 23y | NA | N | N | N | PKD | - | - | - | - |
| 76 | c.710dupA | ex 8 | fr | sporadic | 6y | EU | lip  dysm | tongue  fren | limbs | - | - | - | - | - |
| 89 | c.709_710delAA | ex 8 | fr | sporadic | 6y | NorthA | milia  hair  dysm | tongue  fren  cleft  palate  teeth | limbs  short | - | - | CA | MD  HL | mMI |
| 95 | c.710dupA | ex 8 | fr | sporadic | 5y | EU | milia  hair  dysm | tongue  palate | limbs | - | - | Cy | MD | mMI |
| 110 | c.710delA | ex 8 | fr | sporadic | 35y | EU | dysm  lip | tongue  fren  palate  teeth | N | PKD | - | - | - | - |
| 116 | c.710dupA | ex 8 | fr | familial | 4y | NA | N | tongue  fren | N | - | - | - | - | - |
| *148 | c.710dupA | ex 8 | fr | sporadic | 5y | EU | dysm  lip | tongue  fren  palate | limbs | - | - | - | - | - |
| *150 | c.710dupA | ex 8 | fr | sporadic | 5y | ASIA | milia  dysm | tongue  cleft  palate  teeth | N | - | - | - | - | - |
| S7 | c.675delC | ex 8 | fr | sporadic | 2y | EU | lip  hair  dysm | fren  tongue  palate  teeth | limb  short | - | ACC | - | N | LaD |
| *TH4 | c.710dupA | ex 8 | fr | sporadic | 25y | EU | dysm | tongue  fren  palate  teeth | limb | PKD | ACC | - | N | Bi |
| 6 | c.837_838delAA | ex 9 | fr | familial | 24y | EU | milia  hair  dysm | tongue  fren  palate | limbs | PKD | - | N | N | mMI  ADHD |
| 11 | c.790dupG | ex 9 | fr | sporadic | 20y | EU | N | tongue  palate  teeth | limbs | - | - | ACC | N | N |
| 18 | c.877_878delAT | ex 9 | fr | sporadic | 10y | AU | milia  hair | tongue  fren  palate  teeth | limbs | - | - | - | - | - |
| 43 | c.871A>T | ex 9 | nons | sporadic | 37y | NA | hair  dysm | tongue  fren  teeth | N | - | - | - | - | - |
| 63 | c.877_878delAT | ex 9 | fr | familial | 44y | EU | Lip | tongue  cleft  palate | limbs | - | - | N | N | LeD |
| 63a | c.877_878delAT | ex 9 | fr | familial | 15y | EU | dysm | fren | limbs | PKD | - | ACC | N | mMI |
| 90 | c.837_841delAAAAG | ex 9 | fr | sporadic | 25y | ASIA | dysm | tongue  palate  teeth | limbs  short | PKD | Cy | - | N | Bi |
| 98 | c.839_840delAA | ex 9 | fr | sporadic | 7y | ASIA | dysm  lip | tongue  fren  cleft  palate | limbs | - | ACC  Cy  CA  MCDs | - | Hy | MI |
| 126 | c.877_878delAT | ex 9 | fr | sporadic | 4y | NA | milia | tongue  fren  cleft  palate | limbs | - | - | - | - | - |
| *155 | c. 914_915delAA | ex 9 | fr | sporadic | 19y | EU | milia  hair  dysm | tongue  fren  cleft  palate  teeth | limb  short | - | - | - | - | - |
| S9 | c.895_896insGA | ex 9 | fr | sporadic | 41y | EU | dysm | tongue  teeth | limb | PKD | CDAs  ACC | - | E | MI  Bip |
| S11 | c.919delG | ex 9 | fr | sporadic | 22y | EU | dysm  hair  lip | tongue  fren  teeth | limb | - | CDAs  ACC  Cy  MCDs | - | N | mMI |
| 25 | c.1056C>G | ex 10a | miss | familial | 19y | EU | milia  hair | tongue  palate  teeth | limbs | PKD | - | - | - | - |
| 30 | c.1100G>A | ex 11 | miss | sporadic | 29y | EU | dysm  lip | tongue  fren  teeth | limbs | PKD | - | - | - | - |
| 140 | c.1099C>T | ex 11 | nons | sporadic | 30y | NA | dysm | palate  teeth | limbs | - | - | ACC  CA | HL | mMI |
| *147 | c.1099C>T | ex 11 | nons | sporadic | 3y | EU | N | tongue  fren  cleft  palate | limbs | - | - | - | - | - |
| *151 | c.1128A>G | ex 11 | splice | sporadic | 2y | EU | hair  dysm | tongue  fren  cleft | limbs | - | - | ACC  H | N | N |
| *156 | c.1099C>T | ex 11 | nons | sporadic | 2y | EU | milia  hair | tongue  cleft | limb | - | ACC  MCDs  Cy | - | N | N |
| *159 | c.1059T>A | ex 11 | nons | sporadic | 3y | EU | milia  hair  dysm  lip | tongue  fren  palate  teeth | limb | - | - | - | - | - |
| 35 | c.1130-20_1130-17delAATT | int 11 | splice | familial | 8y | EU | dysm  lip | palate | limbs | - | ACC | - | N | N |
| *TH5 | c.1130-1G>A | int 11 | splice | sporadic | 2y4m | EU | dysm | tongue  fren | limb | - | ACC  CA  CDAs | - | MD | sMI |
| 41 | c.1193_1196delAATC | ex 12 | fr | familial | 7y | EU | dysm | tongue  fren  cleft | limbs | - | - | ACC | N | N |
| 49 | c.1178dupA | ex 12 | fr | sporadic | 26y | EU | N | tongue  fren  teeth | limbs  short | - | - | - | - | - |
| 55 | c.1193_1196delAATC | ex 12 | fr | familial | 9y | EU | dysm | tongue  fren  palate | limbs | - | - | - | - | - |
| 56 | c.1220_1221+1delAGG | ex 12 | fr | sporadic | 49y | AU | N | tongue  fren | N | PKD | - | - | - | - |
| 91 | c.1193_1196delAATC | ex 12 | fr | sporadic | 5y | NorthA | milia  hair  dysm | tongue  fren  teeth | limbs | - | H  MCDs | - | MD | MI |
| 107 | c.1193_1196delAATC | ex 12 | fr | sporadic | 7y | EU | hair  dysm | tongue  fren  palate | limbs | - | - | - | - | - |
| 114 | c.1185delA | ex 12 | fr | sporadic | Dead at 7y | EU | lip  dysm | tongue  fren  palate | limbs | - | ACC  CDAs  (DWM)  MCDs | - | MD | MI |
| *161 | c.1193_1196del  AATC | ex 12 | fr | sporadic | 1y | EU | milia  dysm  lip | tongue  palate | limb | - | - | - | - | - |
| 138 | c.1221+1delG | int 12 | splice | sporadic | 22y | EU | dysm | tongue  fren  cleft  palate  teeth | limbs | PKD | - | N | N | LeD |
| 1 | c.1303A>C | ex 13 | miss | familial | 16y | EU | milia  hair | tongue  teeth | N | - | HH  MCDs | - | He  E | N |
| 20 | c.1268_1272delAAAAC | ex 13 | fr | sporadic | 8y | EU | milia  dysm | tongue  palate | limbs | - | ACC | - | HL | Bi |
| 33 | c.1318delC | ex 13 | fr | sporadic | 8y | NA | milia  dysm | tongue  fren | limbs | - | - | - | - | - |
| 44 | c.1268_1272delAAAAC | ex 13 | fr | sporadic | 9y | EU | milia | tongue  teeth | N | - | - | ACC | cnD | N |
| 60 | c.1319delT | ex 13 | fr | sporadic | 9y | EU | N | tongue  fren | limbs | - | - | - | - | - |
| 112 | c.1323_1326delAGAA | ex 13 | fr | familial | 51y | EU | dysm | fren  palate | limbs | - | - | - | - | - |
| 112a | c.1323_1326delAGAA | ex 13 | fr | familial | Dead at 4y | EU | N | N | N | PKD | - | N | MD | MI |
| 124 | c.1334_1335delTG | ex 13 | fr | sporadic | 4y | NA | Lip | tongue  fren  palate | limbs | PKD | CDAs  (DWM)  ACC  Cy  CA  MCDs | - | N | N |
| 128 | c.1322_1326delAAGAA | ex 13 | fr | sporadic | 4y | EU | dysm | tongue  fren | limbs | - | - | - | - | - |
| S4 | c.1360_1363delCTTA | ex 13 | fr | sporadic | 39y | EU | facial  hair | tongue  fren  palate  teeth | limb | PKD | ACC  Cy | - | N | mMI |
| 27 | c.1452_1458delAGAACTA | ex 14 | fr | sporadic | 25y | AU | dysm  lip | tongue  fren  palate  teeth | limbs | - | - | N | E | mMI |
| 45 | c.1445_1446delTT | ex 14 | fr | sporadic | 7y | AU | dysm | tongue  fren  cleft | limbs | - | - | Por  CDAs | N | N |
| 51 | c.1420C>T | ex 14 | nons | sporadic | 7y | ASIA | milia  hair  dysm  lip | tongue  fren  palate | limbs | - | - | - | - | - |
| 5 | c.2044dupA | ex 16 | fr | familial | 9y | NA | milia | tongue | limbs | - | - | - | - | - |
| 19 | c.2176delC | ex 16 | fr | sporadic | 22y | AU | dysm | tongue  fren  cleft  teeth | limbs | - | - | N | N | MeP |
| 37 | c.2056delT | ex 16 | fr | sporadic | 7y | EU | hair  dysm  lip | tongue  fren  palate | limbs | - | - | - | - | - |
| 57 | c.1979_1980delCT | ex 16 | fr | sporadic | 12y | EU | Lip | tongue  cleft | N | - | - | - | - | - |
| 77 | c.1358T>A | ex 16 | nons | sporadic | 38y | EU | dysm | tongue  fren | limbs | PKD | - | - | - | - |
| 106 | c.1979_1980delCT | ex 16 | fr | sporadic | 6y | AU | dysm | tongue  teeth | limbs | - | ACC  MCDs | - | MD  HL | MI |
| F1 IV 3 | c.2349delC | ex 17 | fr | familial | Fetus | EU | dysm  lip | fren  tongue | limb | - | CDAs  Cy  Por  ACC | - | N | N |
| F2 IV 7 | c.1821delG | ex 16 | fr | familial | 4y2m | EU | dysm  lip | fren  tongue | short | - | ACC  Cy  MCDs | - | E  MD | moMI |
| 82 | c.2261-1G>T | int 16 | splice | sporadic | 6y | ASIA | hair  dysm | tongue  fren  cleft  palate  teeth | limbs | - | - | N | MD  MoCo | Bi |
| 137 | macrodel ex 10-11 | - | - | sporadic | 4y | NA | N | tongue  fren | limbs | PKD | - | - | - | - |
| *139 | macrodel ex 11 | - | - | sporadic | 24y | EU | N | tongue  fren | limbs | - | - | - | - | - |
| *141 | macrodel ex 7-10 | - | - | sporadic | 13y | EU | milia  hair  dysm | tongue  fren  palate | limbs  short | - | ACC  Cy  H | - | N | N |
| F1 III2-08 | macrodel ex 15-23 | - | - | familial | 61y | EU | dysm | tongue  palate | limb  short | PKD | ACC  CDAs | - | cnD  E | mMI |
| F1 IV2-08 | macrodel ex 15-23 | - | - | familial | 37y | EU | dysm  hair | palate  teeth | limb | - | ACC | - | E | MI |
| 3TH-08 | macrodel ex 1-11 | - | - | sporadic | 22y | EU | dysm | tongue  fren  palate | limb  short | PKD | Cy  CA | - | MD | MI |
| Case 6 | macrodel ex 17 | - | - | sporadic | 29y | EU | dysm | fren  tongue  palate  teeth | limb | PKD | ACC | - | E | MI |
|  |  |  |  |  |  |  |  |  |  |  |  |  |  |  |

**LEGEND TO THE TABLE:**

ID 38a = niece of ID 38

ID 63a = daughter of ID 63

ID 74a and ID 74b = twin sisters

ID 112a = daughter of ID 112

* mutations described in the current paper

miss = missense mutation

nons = nonsense mutation

fr = frameshift mutation

splice = splice site mutation

EU = Europe

NA = North America

AU = Australia

ASIA = Asia

NorthA = North Africa

hair = abnormal hair/alopecia (sparse/dry/brittle/coarse hair and/or areas of alopecia)

milia = presence of aberrant milia

dysm = facial dysmorphism (frontal bossing, facial asymmetry, epicanthus, hypertelorism/telecanthus, downslanting palpebral fissures, broad nasal bridge, flattened nasal tip, hypoplasia of the nasal alae, abnormal ears, flat midfacial region, and microretrognathia)

lip = cleft lip/pseudocleft of the upper lip

tongue = tongue anomalies ((bifid/lobulated tongue, tongue lump(s)/hamartoma(s)/lipoma(s) and ankyloglossia)

fren = aberrant oral frenula

cleft = alveolar ridge clefting

palate = cleft palate/high arched palate

teeth = teeth abnormalities (missing/supernumerary teeth, malposition of teeth, enamel hypoplasia)

short = short stature

limb = limb anomalies (forelimb: short hands/brachydactyly, ulnar/radial deviation, broad thumb(s), preaxial polydactyly, duplicated/bifd thumb, mesoaxial polydactyly, postaxial polydactyly; hindlimb: brachydactyly, syndactyly, hallux valgus/broad hallux, preaxial polydactyly, unilateral duplicated/bifd hallux, bilateral duplicated/bifd hallux, mesoaxial polydactyly)

PKD = cystic kidney disease

HH = hypothalamic hamarthoma

MCDs = malformations of cortical development

CA = cerebral atrophy/hypoplasia

ACC = agenesis/hypoplasia of the Corpus Callosum

CDAs = cerebellar developmental anomalies

DWM = Dandy-Walker Malformation

Cy = intracerebral arachnoid cysts

H = hydrocephalus/ventriculomegaly

Por = porencephaly

BrA = brainstem anomalies

He = hemiparesis

E = epilepsy

Hy = hypotonia

Ny = nystagmus

MD = motor delay

HL = sensorineural hearing loss

MoCo = motor coordination deficit

cnD = cranial nerve Disorders

S = spasticity

mMI = mild mental retardation

Bi = borderline intelligence

sMI = severe mental retardation

moMI = moderate mental retardation

MI = mental retardation (severity not specified)

LaD = language disorders

LeD = learning disabilities

MeP = memory problems

ADHD = attention deficit and hyperactivity disorder

Bip = bipolar disorder

N = normal

- = not reported
