# Supplementary material for: CNS involvement in OFD1 syndrome: a clinical, molecular, and neuroimaging study
Source: Orphanet J Rare Dis. 2014 May 10;9:74. doi: 10.1186/1750-1172-9-74 (PMC4113190; doi:10.1186/1750-1172-9-74)
Supplement: Additional file 2: Table S2 — Details of developmental assessment in patients ID50 and ID39. [file 1750-1172-9-74-S2.doc]

Additional file 2: Table S2. Details of developmental assessment in patients ID50 and ID39

| **GMDS (0-8 age)** | | Pt ID50 52 months | | Pt ID39 8 year-old | |
| --- | --- | --- | --- | --- | --- |
| Sub-scale | | SQ^1^ | DA^2^ months | SQ^1^ | DA^2^ months |
| A | **Locomotor** | *42* | *24* | *26.5* | *26* |
| B | **Personal-social** | *61.5* | *32* | *39* | *38* |
| C | **Hearing and Speech** | *73* | *38* | *37* | *36* |
| D | **Eye and hand coordination** | *61.5* | *32* | *35* | *34* |
| E | **Performance** | *50* | *26* | *33* | *32* |
| F | **Practical reasoning** | *73* | *38* | *45* | *44* |
|  | **GQ^3^** | *60* | *31.5* | *36* | *35* |

*Note: GMDS: Griffiths Mental Development Scales; SQ^1^: sub-quotient for sub-scale;* *DA^2^: developmental age for sub-scale,*

*GQ^3^***:** *the General Quotient is obtained by taking the average of all the six sub-quotients*
