# Supplementary material for: CNS involvement in OFD1 syndrome: a clinical, molecular, and neuroimaging study
Source: Orphanet J Rare Dis. 2014 May 10;9:74. doi: 10.1186/1750-1172-9-74 (PMC4113190; doi:10.1186/1750-1172-9-74)
Supplement: Additional file 3: Table S3 — Details of behavioral assessment in patients ID50, ID39 and ID13. [file 1750-1172-9-74-S3.doc]

Additional file 3: Table S3. Details of behavioral assessment in patients ID50, ID39 and ID13

| **CBCL/6-18 years**  (clinical cut-off =70) | Pt. ID50 | Pt. ID39 | Pt. ID13 |
| --- | --- | --- | --- |
| **Anxious-depressed** | *55* | *60* | *60* |
| **Withdrawn/Depressed** | *50* | *55* | ***70*** |
| **Somatic complaints** | *50* | *50* | *59* |
| **Social Problems** | *55* | *56* | *58* |
| **Thought Problems** | *50* | *50* | *50* |
| **Attention Problems** | ***78*** | ***75*** | *51* |
| **Rule-Breaking Behavior** | *53* | *53* | *50* |
| **Aggressive Behavior** | *50* | *50* | *50* |
