# Supplementary material for: CNS involvement in OFD1 syndrome: a clinical, molecular, and neuroimaging study
Source: Orphanet J Rare Dis. 2014 May 10;9:74. doi: 10.1186/1750-1172-9-74 (PMC4113190; doi:10.1186/1750-1172-9-74)
Supplement: Additional file 4: Table S4 — Details of neuropsychological assessment in patient ID13. [file 1750-1172-9-74-S4.doc]

**Verbal**

**memory**

|  |  |  |  |  |  | |  | WISC III | | | |  |  |  |  |  |  | | |  |
| --- | --- | --- | --- | --- | --- | --- | --- | --- | --- | --- | --- | --- | --- | --- | --- | --- | --- | --- | --- | --- |
|  | **Subtest** | **score** (mean of 10 and a standard deviation of 3) | | | | | | | | | | |  |  | **IQ or Index Quotient** | | | | | |
|  |  |  |  |  |  | |  |  |  |  | |  |  |  |  | **scores** |  | | |  |
|  |  |  |  |  |  | |  |  |  |  | |  |  |  | Mean= 100; standard | | | | | |
|  |  |  |  |  |  | |  |  |  |  | |  |  |  |  | deviation = 15. | | | | |
| **Verbal**  **subtest** | **Information** |  | 8 | **Performance**  **Subtests** | | |  | **Picture Completion** | | | | | 7 | | **FSIQ** |  |  | | 85 | |
|  |  |  |  |  |  |  |  |  |  |  |  |  |  |  |  |  |  |  |  |  |
|  | **Similarities** |  | 7 |  | | |  | **Coding** | |  | |  | 11 | | **VIQ** |  |  | | 77 | |
|  |  |  |  |  |  |  |  |  |  |  |  |  |  |  |  |  |  |  |  |  |
|  |  |  |  |  |  |  |  |  |  |  |  |  |  |  |  |  |  |  |  |  |
|  | **Arithmetic** |  | 6 |  | | |  | **Picture Arrangement** | | | | | 10 | | **PIQ** |  |  | | 94 | |
|  |  |  |  |  |  |  |  |  |  |  |  |  |  |  |  |  |  |  |  |  |
|  |  |  |  |  |  |  |  |  |  |  |  |  |  |  |  |  |  |  |  |  |
|  | **Vocabulary** |  | 6 |  | | |  | **Block Design** | | | |  | 8 | | **VCI** |  |  | | 79 | |
|  |  |  |  |  |  |  |  |  |  |  |  |  |  |  |  |  |  |  |  |  |
|  |  |  |  |  |  |  |  |  |  |  |  |  |  |  |  |  |  |  |  |  |
|  |  |  | 6 |  | | |  |  |  |  | |  | 11 | |  |  |  | | 90 | |
|  | **Comprehension** | |  |  |  | |  | **Object Assembly** | | | | |  |  | **POI** |  |  | |  |  |
|  | **Digit Span** |  |  |  |  | |  | **Symbol Search** | | | | | 12 | | **FDI** |  |  | | 91 | |
|  |  |  |  |  |  | |  | **Mazes** | |  | |  | 8 | | **PSI** |  |  | | 109 | |
|  | **TOMAL Subtest Score** (mean of 10 and a standard deviation of 3)  **Non verbal**  **memory** | | | | | | | | | | | | | | |  |  | |  | |
|  | **Word Selective Reminding** | |  |  |  | |  |  |  | | **Spatial memory** | | |  |  |  |  | | 9 | |
|  |  |  |  |  |  |  |  |  |  |  |  |  |  |  |  |  |  |  |  |  |
|  | **Word Selective Reminding Delayed** | | |  |  | | 12 | |  |  | **Visual Selective Reminding** | | | | |  |  | |  | |
|  |  |  |  |  |  |  |  |  |  |  |  |  |  |  |  |  |  |  |  |  |
|  | **Memory for Stories** | | | | | |  | |  |  | **Visual Selective Reminding Delayed** | | | | | |  | | 11 | |
|  |  |  |  |  |  |  |  |  |  |  |  |  |  |  |  |  |  |  |  |  |
|  |  |  |  |  |  |  |  |  |  |  |  |  |  |  |  |  |  |  |  |  |
|  | **Memory for Stories Delayed** | | |  |  | |  | 6 |  |  |  |  |  |  |  |  |  | |  |  |
|  |  |  |  |  |  |  |  |  |  |  |  |  |  |  |  |  |  |  |  |  |
|  |  |  |  |  |  |  |  |  |  |  |  |  |  |  |  |  |  |  |  |  |
| **VMI: Index Quotient scores** (Mean = 100; standard deviation = 15)  scores  Mean = 100; standard deviation = 15) | | | | | | | | | | | | | | | | |  | |  | |
| **VMI index** | |  |  |  |  | |  | 94 | **Visual index** | | | |  |  |  |  |  | | 108 | |
|  |  | **WCST** (Mean = 100; standard deviation = 15) | | | | | | | | | | | |  |  |  |  | |  | |
| **No-perseverant error** | |  |  |  |  | | 90 | | **perseverant error** | | | |  |  |  |  |  | | 66 | |
|  |  | **Verbal fluency** (Mean = 10; standard deviation = 3) | | | | | | | | | | | | |  |  |  | |  | |
| **phonological categories** | | | | | |  | | | **semantic categories** | | | | | | | | |  | | |

Additional file 4: Table S4. Details of neuropsychological assessment in patient ID13

11

11

10

9

6

6

**Verbal**

**subtest**

**Performance**

**Subtests**

**Non verbal**

**memory**

Verbal comprehension Index (VCI)

Perceptual Organization Index (POI)

Freedom from Distractibility Index (FDI)

Processing Speed Index (PSI)

Wisconsin Card Sorting Test (WCST)
